# Supplementary figures and images for: Flexibility to contingency changes distinguishes habitual and goal-directed strategies in humans
Source: PLoS Comput Biol. 2017 Sep 28;13(9):e1005753. doi: 10.1371/journal.pcbi.1005753 (PMC5634647; doi:10.1371/journal.pcbi.1005753)

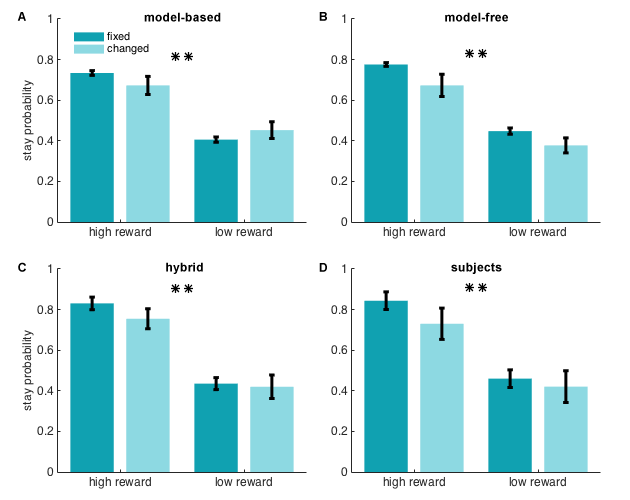

Supplement: S1 Fig — Stay probability patterns after first-level contingency changes predicted by simulating model-based (A), model-free (B), and hybrid (C) reinforcement learning algorithms, along with experimental results (D). Stay-probability measures the probability of choosing the first-level action that results in the same second-level state as the previous trial, following a trial that started at the first level. For each system, this index was measured under four different conditions: when the reward received in the previous trial was “high” or “low”, and when the transition experienced in the previous trial (relative to the trial before that) “changed” or remained “fixed”. * p < 0.05, ** p < 0.01. (TIF) [file pcbi.1005753.s001.tif]

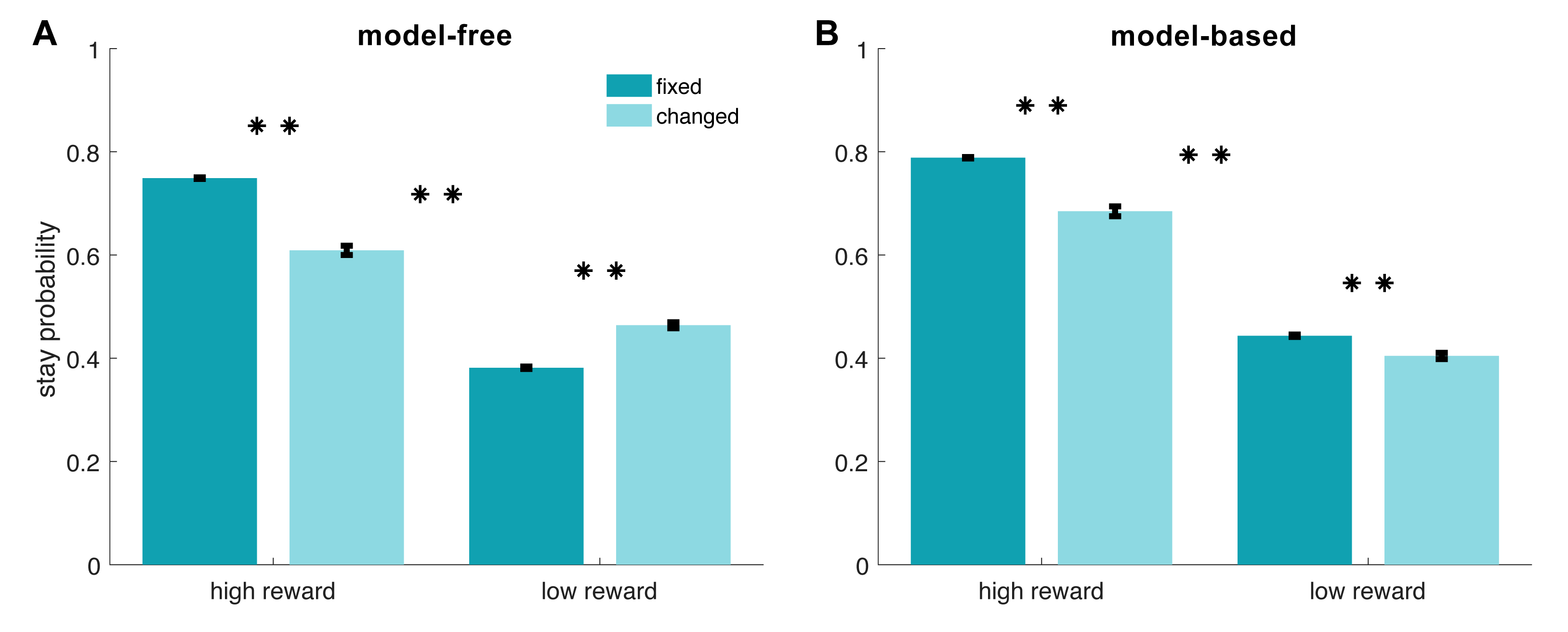

Supplement: S2 Fig — Stay probability patterns after first-level contingency changes predicted by simulating model-free (A) and model-based (B) algorithms for 1000 agents. When starting from the first level and encountering a change in the transition structure, both MB and MF systems are able to update their action values. However, the extent of this update is not equal for the two systems due to their different effective learning rates. As a result, the two systems show slightly different flexibility levels (i.e., stay-probability patterns) even when both systems were informed of the change in contingencies. Therefore, in addition to structural differences, the MB and MF algorithms that participants used in this task also have different effective learning rates. This difference is not apparent in S1 Fig because the models were only simulated 16 times (equal to the number of participants). (TIF) [file pcbi.1005753.s002.tif]

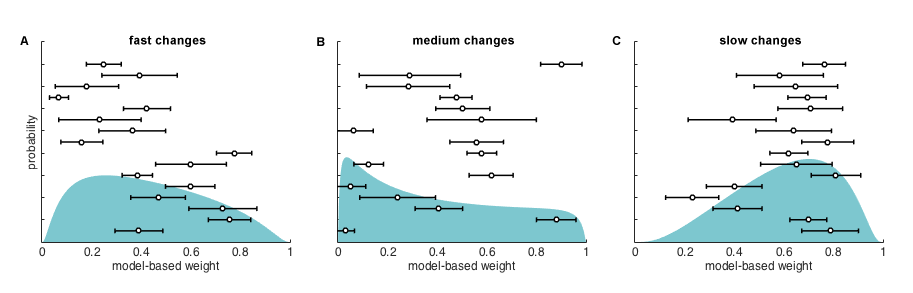

Supplement: S3 Fig — Model-based weights for (A) fast, (B) medium and (C) slow contingency changes. Probability density function over the model-based weight parameters estimated from model-fitting, for the blocks of fast (every 3–6 trials), medium (every 7–10 trials) and slow (every 11–14 trials) frequency of contingency changes. Overlaid are the individual subjects’ parameter estimates for each block type. Error bars represent standard deviation. (TIF) [file pcbi.1005753.s003.tif]

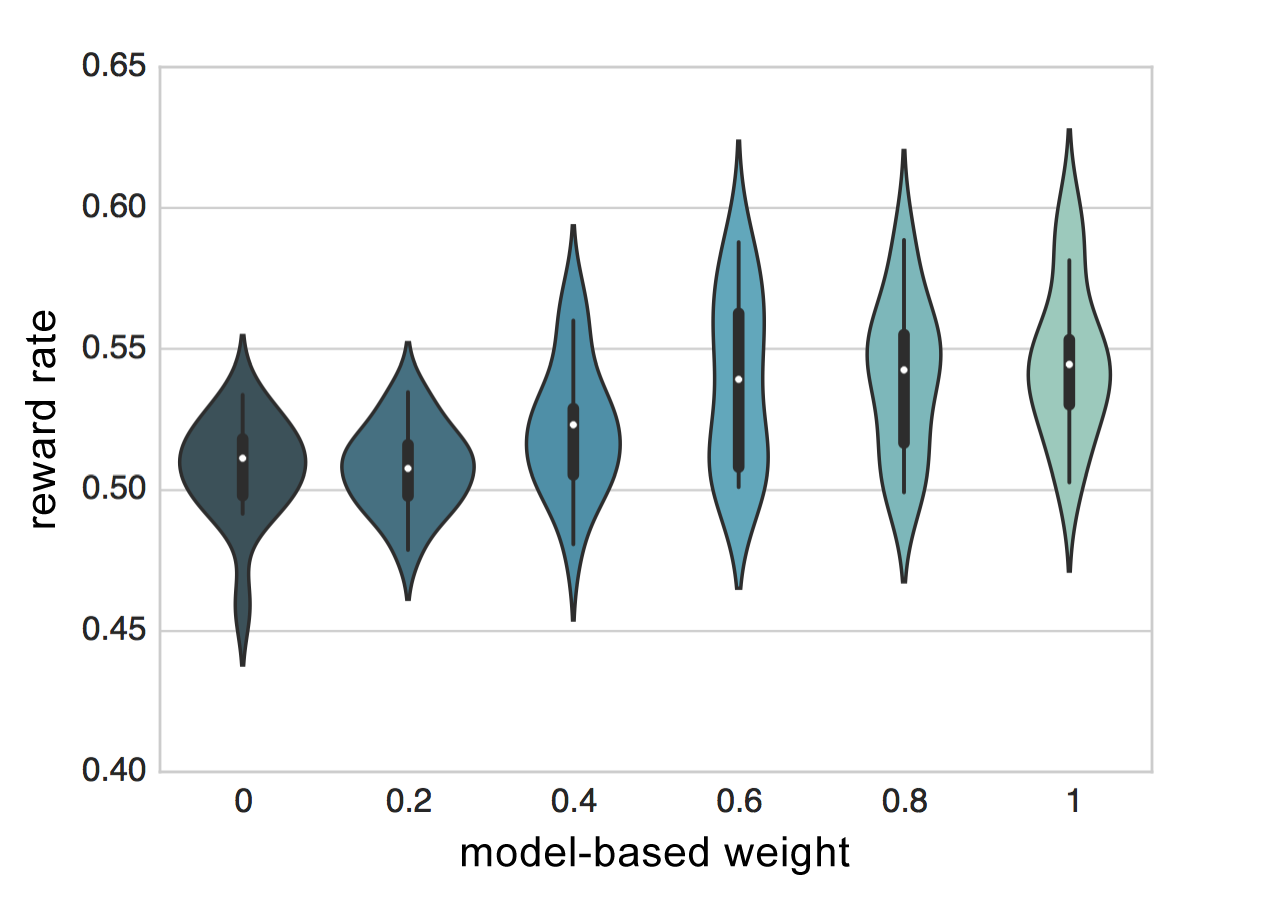

Supplement: S4 Fig — Choices were simulated with six different model-based weights (0, 0.2, 0.4, 0.6, 0.8, 1, with n = 16 iterations each) and the mean reward rate was computed. There was a significant difference in reward rate across different wMB values, F(5,90) = 8.5, p < 0.01, however, the difference was small, which may account for the absence of a significant modulation in wMB across contingency change frequencies. (TIF) [file pcbi.1005753.s004.tif]
