# Supplementary material for: Flexibility to contingency changes distinguishes habitual and goal-directed strategies in humans
Source: PLoS Comput Biol. 2017 Sep 28;13(9):e1005753. doi: 10.1371/journal.pcbi.1005753 (PMC5634647; doi:10.1371/journal.pcbi.1005753)
Supplement: S1 Table — Best-fitting parameter estimates over the subjects from model-fitting. (DOCX) [file pcbi.1005753.s005.docx]

**S1 Table. Median Plus Quartile Group-level Parameter Estimates.**

|  | ***β*** | **Stay bias** | ***α_MB_*** | ***α_MF_*** | ***λ*** | ***wMB***  **(block 1)** | ***wMB***  **(block 2)** | ***wMB***  **(block 3)** |
| --- | --- | --- | --- | --- | --- | --- | --- | --- |
| **1st quartile** | 1.88 | 0.04 | 0.55 | 0.03 | 0.30 | 0.10 | 0.40 | 0.48 |
| **Median** | 2.99 | 0.10 | 0.70 | 0.30 | 0.47 | 0.23 | 0.57 | 0.63 |
| **3rd quartile** | 4.73 | 0.22 | 0.81 | 0.85 | 0.65 | 0.46 | 0.71 | 0.76 |
