# Supplementary material for: Flexibility to contingency changes distinguishes habitual and goal-directed strategies in humans
Source: PLoS Comput Biol. 2017 Sep 28;13(9):e1005753. doi: 10.1371/journal.pcbi.1005753 (PMC5634647; doi:10.1371/journal.pcbi.1005753)
Supplement: S2 Table — Integrated Bayesian Information Criterion (iBIC) and negative log-likelihood of all candidate models from model-fitting. The models tested were: pure model-free (“MF”), pure model-based (“MB”), hybrid MB/MF (“hybrid”), hybrid MB/MF with different weights fitted for each of the three 200-trial blocks (“three-block hybrid”), and a hybrid model with different weights fitted for each frequency of contingency changes (“three-frequency hybrid”). The winning model was the three-block hybrid, highlighted in gray, according to iBIC [24] and Bayesian model selection [25]. (DOCX) [file pcbi.1005753.s006.docx]

**S2 Table. Model Comparison of Candidate Models.**

| **Model** | **Model-free** | **Model-based** | **Hybrid** | **Three-block hybrid** | **Three-frequency hybrid** |
| --- | --- | --- | --- | --- | --- |
| **Parameters** | 4 | 3 | 6 | 8 | 8 |
| **iBIC** | 9051 | 8091 | 7938 | 7687 | 7711 |
| **Negative Log Likelihood** | 4489 | 4018 | 3914 | 3770 | 3782 |
