# Supplementary material for: Flexibility to contingency changes distinguishes habitual and goal-directed strategies in humans
Source: PLoS Comput Biol. 2017 Sep 28;13(9):e1005753. doi: 10.1371/journal.pcbi.1005753 (PMC5634647; doi:10.1371/journal.pcbi.1005753)
Supplement: S3 Table — Integrated Bayesian Information Criterion (iBIC) and negative log-likelihood of the winning three-block hybrid model with different weights fitted for each of the three 200-trial blocks and the same model without stay bias, with λ = 1, with λ = 0, with only one learning rate for both MF and MB systems, and without updating fictive reward. The full model fit better to the data than the same model without each of the aforementioned parameters, even when controlling for model complexity in the iBIC. (DOCX) [file pcbi.1005753.s007.docx]

**S3 Table. Model Comparison of Additional Parameters.**

| **Model** | **Full model** | **No stay bias** | ***λ* = 1** | ***λ* = 1** | **One learning rate** | **No fictive reward** |
| --- | --- | --- | --- | --- | --- | --- |
| **Parameters** | 8 | 7 | 7 | 7 | 7 | 8 |
| **iBIC** | 7687 | 8047 | **7703** | 7754 | 7774 | 8024 |
| **Negative Log Likelihood** | 3770 | 3959 | 3787 | 3813 | 3823 | 3939 |
